# NEW PERSPECTIVES ON EXPLICIT AND IMPLICIT AGEIST DISCOURSES IN DESIGNING DIGITAL TECHNOLOGIES FOR OLDER PERSONS

**DOI:** 10.1093/geroni/igae098.1553

**Published:** 2024-12-31

**Authors:** Ittay Mannheim, Eveline Wouters, Hanna Köttl, Leonieke Van Boekel, Rens Brankaert, Yvonne van Zaalen

**Affiliations:** Ben Gurion University of the Negev, Beer-Sheva, HaDarom, Israel; Fontys University of Applied Science, Eindhoven, Noord-Brabant, Netherlands; IMC University of Applied Sciences Krems, Krems an der Donau, Wien, Austria; SOVAK, Terheijden, Noord-Brabant, Netherlands; Eindhoven University of Technology, Eindhoven, Noord-Brabant, Netherlands; The Hague University Of Applied Sciences, The Hague, Zuid-Holland, Netherlands

## Abstract

Involving older persons throughout the design process of digital technology (DT) is considered a best practice for designing beneficial and acceptable DTs. Nevertheless, negative discourses on aging and ageism are potential underlying factors that could influence which and how DTs are designed and how older persons are involved in the design process. This presentation reflects on previous findings from a scoping review on the explicit and implicit manifestations of ageism in the design process of DT and demonstrates their relevance in new findings. Seven databases were screened for studies reporting on the design of DT with older persons. Sixty articles met the inclusion criteria. Information regarding the study and the discourse about older persons and their involvement in the design process of DT was coded and analyzed using critical discourse analysis. Various explicit and implicit manifestations of ageism, forms of exclusion, errors, and biases in designing DT with older persons were identified. The critical discourse analysis revealed the use of outdated language, stereotypical categorizations, and design decisions based on ageism. Generally, a discrepancy was found between an ‘ideal’ discourse regarding the involvement of older persons throughout the design process and actual practice. Notably, while the findings of the scoping review focused on publications until January 2020, recent research regarding the design process of DT is reflected upon using these previous findings. Lessons learned for mitigating ageism and promoting meaningful inclusion of older persons in the design process of DT are discussed.

